# Convolutional neural networks decode finger movements in motor sequence learning from MEG data

**DOI:** 10.3389/fnins.2025.1623380

**Published:** 2025-09-09

**Authors:** Aleksey Zabolotniy, Russell Weili Chan, Victoria Moiseeva, Tommaso Fedele

**Affiliations:** ^1^Institute of Cognitive Neuroscience, National Research University Higher School of Economics, Moscow, Russia; ^2^Department of Learning, Data Analytics and Technology, Section Cognition, Data and Education, Faculty of Behavioural, Management and Social Sciences, University of Twente, Enschede, Netherlands

**Keywords:** finger movement decoding, MEG, convolutional neural networks, serial reaction time task, motor learning

## Abstract

**Objective:**

Non-invasive Brain–Computer Interfaces provide accurate classification of hand movement lateralization. However, distinguishing activation patterns of individual fingers within the same hand remains challenging due to their overlapping representations in the motor cortex. Here, we validated a compact convolutional neural network for fast and reliable decoding of finger movements from non-invasive magnetoencephalographic (MEG) recordings.

**Approach:**

We recorded healthy participants in MEG performing a serial reaction time task (SRTT), with buttons pressed by left and right index and middle fingers. We devised classifiers to identify left vs. right hand movements and among four finger movements using a recently proposed decoding approach, Linear Finite Impulse Response Convolutional Neural Network (LF-CNN). We also compared LF-CNN to existing deep learning architectures such as EEGNet, FBCSP-ShallowNet, and VGG19.

**Results:**

Sequence learning was reflected by a decrease in reaction times during SRTT performance. Movement laterality was decoded with an accuracy superior to 95% by all approaches, while for individual finger movement, decoding was in the 80–85% range. LF-CNN stood out for (1) its low computational time and (2) its interpretability in both spatial and spectral domains, allowing to examine neurophysiological patterns reflecting task-related motor cortex activity.

**Significance:**

We demonstrated the feasibility of finger movement decoding with a tailored Convolutional Neural Network. The performance of our approach was comparable to complex deep learning architectures, while providing faster and interpretable outcome. This algorithmic strategy holds high potential for the investigation of the mechanisms underlying non-invasive neurophysiological recordings in cognitive neuroscience.

## 1 Introduction

Decoding human brain activity is crucial for understanding and enhancing motor learning outcomes, as it provides insights into the neural mechanisms underlying movement control, skill acquisition, and adaptation. Brain signal patterns are associated with motor planning, execution, and learning, enabling the development of advanced neurorehabilitation strategies and personalized training protocols. For instance, studies have demonstrated that neurons in the primary motor cortex convey more information about movement direction after learning, indicating improved neuronal coding post-training (Li et al., 2001). Decoding models are also instrumental in translating neuronal group activities into understandable stimulus features, such as stimulus orientation, which is essential for interpreting complex visual scenes or abstract semantic information (Mathis et al., 2024). This knowledge is valuable for individuals recovering from neurological disorders, athletes seeking to optimize performance, and broader applications like brain-computer interfaces (BCI).

Achieving optimal performance in BCI systems requires a careful balance between interdependent factors of speed and accuracy when employing compact convolutional neural networks (CNNs) like EEGNet. While EEGNet often reports of improved accuracy over other CNNs (de Oliveira and Rodrigues, 2023; Rao et al., 2024), the computational demands associated with deep learning models can impact real-time decoding speed and hamper timely feedback in motor learning and control applications. Therefore, optimization of architecture and implementation of efficient computational CNN strategies are essential to maintain a favorable balance between speed and accuracy for practical BCI applications. Recently, a promising method called Linear Finite Impulse Response Convolutional Neural Network (LF-CNN) (Zubarev et al., 2019) emerged as a promising approach to substantially reduce CNN model complexity while preserving high accuracy and interpretability. However, the LF-CNN has only been tested in motor imagery tasks and must yet be validated in the context of more complex motor sequence learning task. The translation and decoding of finger-movements can provide essential information on the brain’s motor learning processes that can be applied to understand mechanisms of motor action, skill mastery, rehabilitation and training.

Understanding how the brain encodes and adapts during motor learning behavior is one of the main challenges in cognitive neuroscience. A key objective is to decompose complex, multidimensional brain data into interpretable representations, unveiling cognitive processes in motor learning (Bijsterbosch et al., 2020). One widely used paradigm to study implicit motor learning is the Serial Reaction Time Task (SRTT), which involves a sequence of key presses in response to spatially mapped visual stimuli (Nissen and Bullemer, 1987; Robertson, 2007). The stimuli appear random and follow a repeating 12-key sequence, making it difficult for participants to identify clear start or end points. As learning progresses, responses typically shift from slow, stimulus-driven reactions to faster, more accurate movements guided by an internalized sequence representation (Chan et al., 2018; O'Connor et al., 2022). The SRTT is particularly well-suited for investigating the neural correlates of motor learning, as it elicits well-characterized changes in cortical activity. Early learning stages are associated with increased engagement of frontal and occipital regions (Albouy et al., 2015), while later stages show a shift toward motor cortex dominance as the sequence becomes more ingrained (Immink et al., 2019; Verwey et al., 2019). These dynamics make the SRTT a valuable tool for validating cortical decoding approaches and systematically examining the neurophysiological mechanisms underlying skill acquisition (Keele et al., 2003). In this study, we recorded magnetoencephalography (MEG) data during SRTT performance to evaluate the capacity of decoding models to capture learning-related changes in cortical activity.

Decoding brain activity associated with hand and finger movements is pivotal in advancing BCI technologies, with essential advances needed for accurate interpretation of neural signals. Recent advances in neural decoding have increasingly emphasized the importance of interpretable deep learning models for capturing fine-grained motor information from non-invasive recordings. For example, Borra et al. (2023) demonstrated that convolutional neural networks can effectively decode movement kinematics from EEG while preserving interpretability, a critical feature for clinical applications. Similarly, Tian et al. (2025) and Jain and Kumar (2025) proposed regression-based and source-imaging approaches to estimate upper limb trajectories and grasp-lift dynamics, respectively, highlighting the growing interest in decoding continuous motor parameters and skill development. Borràs et al. (2025) further emphasized the role of repetition in modulating motor cortical activity, reinforcing the need for models that can adapt to dynamic neural states. Compact CNNs such as those proposed by Cui et al. (2022) and Petrosyan et al. (2021) have shown promise in cross-subject decoding tasks, underscoring the potential of lightweight architectures for real-world BCI deployment.

MEG-based studies have begun to demonstrate the feasibility of decoding complex motor behaviors with high temporal resolution. For instance, Lévy et al. (2025) used MEG to decode sentence-level typing behavior, revealing hierarchical neural dynamics that span motor and cognitive processes. Their Brain2Qwerty model achieved a peak accuracy of ~74% from MEG signals, narrowing the gap between invasive and non-invasive BCI approaches. In another instance, Bu et al. (2023) developed MEG-RPSnet, a convolutional neural network designed to classify hand gestures (rock, paper, and scissors) using MEG data. Using data from 12 participants, the authors combined a tailored preprocessing pipeline with a deep learning architecture trained on single-trial and the model achieved an average classification accuracy of 85.6%, outperforming most traditional machine learning methods and EEG-based neural networks. Notably, the model maintained high performance even when restricted to central-parietal-occipital or occipitotemporal sensor regions, highlighting the spatial specificity of gesture-related neural activity. These recent studies underscore the potential of MEG for decoding fine motor actions such as finger movements and gestures and support the development of interpretable models that can bridge decoding performance with neuroscientific insight.

Despite advances in non-invasive BCIs, decoding individual finger movements using EEG remains challenging due to overlapping cortical representations (Alazrai et al., 2019; Gannouni et al., 2020), noise (Nam et al., 2008), artifacts (Guarnieri et al., 2018), and inter-individual variability (Allison et al., 2010; Volosyak et al., 2011). These factors often can degrade decoding accuracy and obscure causal relationships in neural signals (Mitra and Pesaran, 1999). Recent work by Kim et al. (2023) successfully identified the specific cerebral cortices involved in processing acceleration, velocity, and position during directional reaching movements using MEG data and time-series deep neural network models. This approach not only facilitated the accurate decoding of kinematic parameters, but also utilized explainable AI to pinpoint the distinct and shared cortical regions responsible for each kinematic attribute, providing valuable insights into the neural underpinnings of motor control. Together, these studies underscore the value of MEG for decoding complex motor learning and support the development of interpretable, high-resolution models for gesture-based BCI applications.

Deep learning (DL) has emerged as a powerful solution to many of these challenges by integrating feature extraction and classification into a unified framework, reducing the need for manual data engineering (Mahmud et al., 2018). DL models still offer improved performance but often demand large datasets and high computational resources (Gutierrez-Martinez et al., 2021; Rashid et al., 2020; Saha et al., 2021; Zhang et al., 2021), limiting their use in time-sensitive applications like real-time rehabilitation feedback for BCI applications (Yeager et al., 2016). Effective BCIs must therefore balance accuracy, adaptability, and efficiency for these systems to translate neural signals into timely control commands for assistive technologies (King et al., 2018).

Architectures such as FBCSP ShallowNet (Amin et al., 2019; Schirrmeister et al., 2017) and EEGNet (Al-Saegh et al., 2021; Gemein et al., 2020; Lawhern et al., 2018) have demonstrated strong performance in motor imagery classification, with EEGNet in particular showing high generalizability across paradigms and tasks. Recent work has shown that EEGNet can decode individual finger movements with promising accuracy (Rao et al., 2024). Similarly, the VGG19 architecture, originally developed for image classification, has been adapted for EEG decoding due to its ability to capture complex spatiotemporal features (Bagherzadeh et al., 2023; Simonyan and Zisserman, 2015; Vignesh et al., 2023).

Among these models, the Linear Finite Impulse Response Convolutional Neural Network (LF-CNN) introduced by Zubarev et al. (2019) stands out for its interpretability. Unlike conventional CNNs, LF-CNN employs fully linear spatial and temporal filters, allowing direct visualization of learned weights and facilitating neuroscientific insight. It has demonstrated competitive accuracy in motor imagery tasks while offering enhanced transparency and generalizability. Its lightweight design and interpretability make it particularly suitable for clinical applications where explainability is essential.

In the current study, we aimed to investigate the applicability of the LF-CNN beyond motor imagery by using MEG recordings of motor learning. We applied a two-layer LF-CNN to classify finger movements during the SRTT at the single-trial level. We hypothesized that LF-CNN would be capable of accurately decoding individual finger movements and that its performance would be comparable to more complex deep learning architectures. Additionally, we expected the model to offer faster computational times and interpretable patterns across spatial, temporal, and spectral domains, making it a promising tool for both research and clinical applications.

## 2 Methods and materials

### 2.1 Participants

Eight right-handed subjects (4 females, mean age = 25 years, SD = 6.8) participated to the study. Eligibility criteria included no severe health issues, psychological, neurological, or medical disorders, with a normal vision. The study was conducted according to the Institutional and Ethical Review Board (IRB) guidelines and regulations of the Higher School of Economics. All participants signed the informed consent form and participation agreement.

### 2.2 Experimental protocol

**Figure 1:**
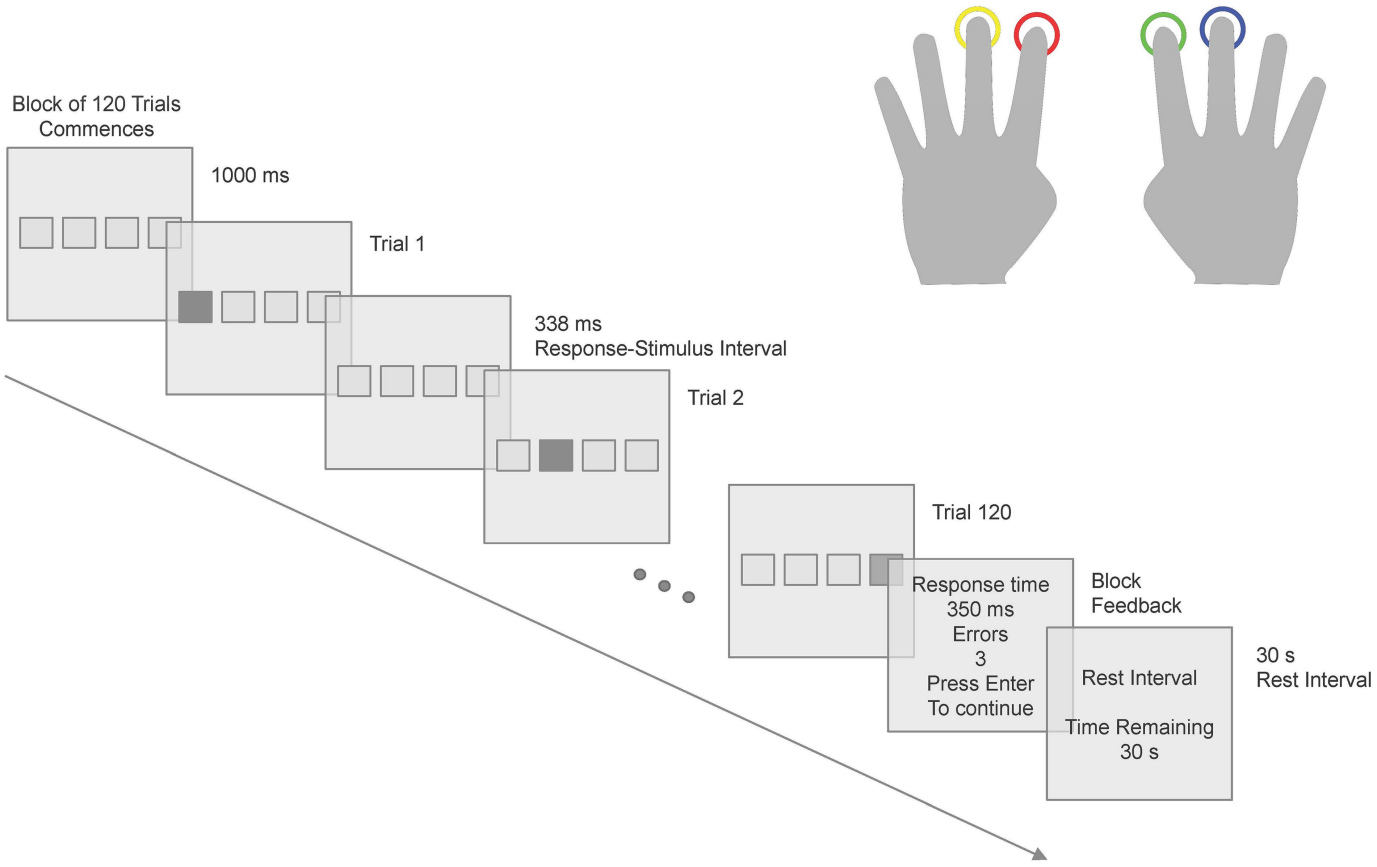
Scheme of one block of the SRTT. The stimulus presentation and subsequent responses followed a specific pattern across 12 learning blocks of the SRTT.

The Serial Reaction Time Task (SRTT) stimuli consisted of a series of four empty boxes, which were spatially mapped to four response keys in that participants used their left middle finger for Key 1 and left index finger for Key 2, right index finger for Key 3 and right middle finger for Key 4 (see Figure 1) on a color-coded controller pad. Participants responded when one of the four boxes turned red. The monitor was set to 64-bit color and had a screen refresh rate of 120 Hz. The participants were sitting in the MEG chair at 1.5 m from the monitor. Each box had a visual angle dimension of 2° × 2°, with a separation of 2° between each box. Following each response, there was a 338 ms interval before the next stimulus was presented. The SRTT consisted of a 12-item sequence (121342314324) repeating for 10 cycles for 12 learning blocks - 120 keypress trials per block, 1,440 keypress trials in total. After each block, participants received performance feedback, including the average reaction time and the number of error trials in that block. Following the feedback presentation, a rest interval of 30 s was given to the participants. The structure of the SRTT is depicted in Figure 1. The SRTT experiment was implemented in E-Prime® 2.0 Software (Psychology Software Tools Inc., Sharpsburg, USA). Participants were presented with on-screen written instructions for the SRTT and also explicitly told that the participants should use the designated fingers to press the corresponding response keys, prioritizing both speed and accuracy. The instructions emphasized that achieving a balance between response speed and accuracy was crucial for optimal performance.

### 2.3 MEG data recording and preprocessing

MEG data was recorded using the Vectorview Neuromag system, which comprised 306 channels including 204 gradiometers and 102 magnetometers, at a sampling frequency of 1,000 Hz. Data were then downsampled at 200 Hz. For subsequent analysis, only the gradiometer data was utilized. To mitigate artifacts, like eye-blinks and heartbeats, an independent component analysis (ICA) was performed. Trials affected by muscle artefacts (Muthukumaraswamy, 2013) were removed by visual inspection resulting in rejection of 5.7 ± 2.5% trials per participant. Following artifact removal, a high-pass finite impulse response (FIR) filter with a cutoff frequency of 0.5 Hz was applied to the signals to prevent data drift.

### 2.4 Data analysis

#### 2.4.1 Data preparation

The MEG continuous data were sorted in epochs of ± 500 ms around the button press (1 trial): movement of left index finger, left middle finger, right index finger and right middle finger. Only trials with correct responses were considered for further analysis. These data underwent 4 classification tasks:

1. Left hand versus right hand (2 classes: left index and left middle is class 1; right index and right middle is class 2),
2. Classification within left hand (2 classes: left index is class 1; left middle is class 2),
3. Classification within right hand (2 classes: right index is class 1; right middle is class 2)
4. 4-fingers classification (4 classes: left index is class 1; left middle is class 2; right index is class 3; right middle is class 4).

Prior to the decoder training procedure, the data were z-scored in each epoch with respect to the mean and standard deviation computed across all channels. We present the analysis of response-locked trials rather than stimulus-locked to investigate the anticipation of the motor response. This is motivated by the fact that initial responses are triggered by visual input in the first blocks of the task (referred as *learning phase*), and anticipated by accumulated knowledge in the last blocks of the SRTT (referred as *learned phase*). For completeness, we included the outcome of four fingers decoding of the stimulus-locked trials in Supplementary Table S7.

#### 2.4.2 Data decoding

**Figure 2:**
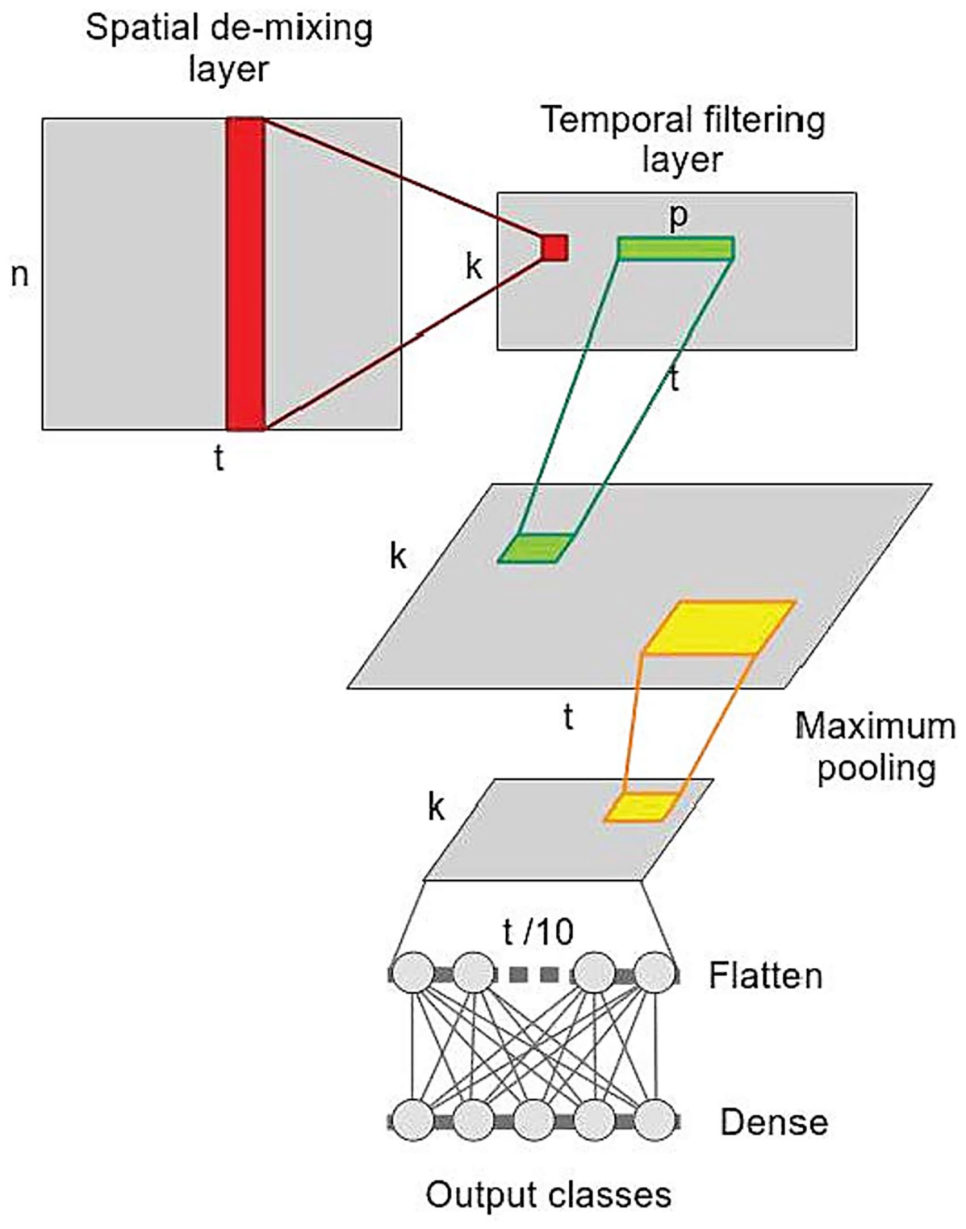
Linear-Finite-Impulse-Response Convolution Neural Network (LF-CNN). n, number of observation (MEG channels), k, number of extracted components, t, time (epochs length), p, length of temporal convolutional filters in the second layer.

The generative model for MEG data can be formulated as in Equation 1


(1) $$X_{t}={\mathrm{AS}}_{t}+\varepsilon$$

where $X_{t}$ is the data observed at the sensor level at the timepoint t, $S_{t}$ are underlying sources projecting their activity to the sensors through the mixing matrix A, and *ε* is Gaussian white observation noise. Regression-based decoders are models that relate stimulus to encoded neural information. The basic principle of regression decoding (Warren et al., 2016) is presented in the computing of a matrix of spatial filters (demixing matrix), such that:


(2) $${\hat{S}}_{t}=W^{T}\cdot X_{t}$$

where W is the demixing matrix, which allows retrieving the estimated sources ${\hat{S}}_{t}$ from the data $X_{t}$ . The demixing matrix W of Equation 2 can be computed with some optimization technique that aims to minimize predetermined cost function (for example stochastic gradient decent) to extract components containing the largest possible amount of information relating the underlying neural activity with the recorded data. Importantly, we emphasize that the matrix W is not itself a representation of such neural activity, but just a transfer function enhancing source related components and suppressing noise related components. To reconstruct the activation pattens mimicking the source of interest, we adopted the methodology described in Haufe et al. (2014):


(3) $$A=K_{\mathrm{xx}}WK_{\mathrm{ss}}^{-1}$$

where A represents the activation patterns, while K_xx_ represent covariance matrices of data in sensor space and K_ss_ is a covariance matrix of feature space, respectively. In our approach, the demixing matrix is obtained in two steps, decomposing and reconstructing the observed data in the spatial and temporal dimension. To this aim, we validated the performance Linear-Finite-Impulse-Response Filtering Convolutional Neural Network (LF-CNN) (Zubarev et al., 2019) on our SRTT dataset. The neural network consists only of 4 layers (Figure 2): (1) a spatial demixing layer applying k filters of size [n × 1] (red) to the input data of shape [n × t], producing latent sources [t × k]; (2) a temporal-filtering layer convolving k filters of size [p × 1] (green) to k latent sources extracted from the spatial demixing layer, producing a temporal filtering output of size [k × t]; (3) a temporal pooling layer (yellow) decreasing the temporal resolution by a factor of 10; (4) a fully connected layer with softmax activation to make prediction. Details on the hyperparameters of the LF-CNN are in Supplementary Table S3.

The spatial demixing layer allows deriving spatially interpretable patterns. Weights estimated by the temporal-filtering layer can be considered as finite-impulse-response filters applied to spatial features, providing insights into the spectral and temporal properties of the extracted components as described in Petrosyan et al. (2021).

#### 2.4.3 Results visualization

In the results section, we propose representative patterns for each set of decoders obtained, showing:

1. Spatial patterns, obtained in accordance to Equation 3 (Haufe et al., 2014), with W being the weights obtained in the first layer of the LF-CNN. These two elements are depicted in the upper panels of Figures in sections 3.2–3.3.

2. Temporal patterns. Obtained by convolving the output of the spatial filtering layer with the temporal filter weights (LF-CNN second layer). After applying the convolution separately to each trial, we averaged the time-resolved signal and also computed the average of the single-trial time–frequency representation. These two elements are superimposed in the middle panels of Figures in sections 3.2–3.3.

3. Spectral representation of input, transfer function and output of the second layer. The frequency spectra of the input to the temporal filter in the second layer (blue), the frequency spectra of the temporal filter convolution kernel, or filter response (filter green), and the frequency spectra of the output of the second layer (orange) are displayed in the bottom panels of Figures in sections 3.2–3.3.

#### 2.4.4 Cross validation schema

To rigorously assess model performance, we adopted a 6-fold split strategy. In each iteration, five folds (83.3%) were used for training, while the remaining fold (16.6%) served as the held-out test set. Within the training phase, we further applied a 5-fold cross-validation on the training portion to create dynamic validation sets. This nested validation approach allowed for continuous monitoring and fine-tuning of the model’s generalization capability across training runs.

This strategy was used to train a decoder for each individual subject. The number of trials used in each subject for training and test are listed in Supplementary Table S2.

We benchmarked this approach with less interpretable deep-learning architectures: Shallow Filter-Bank Common Spatial Patterns CNN (FBCSP-CNN) (Schirrmeister et al., 2017), EEG-Net (Lawhern et al., 2018), VGG-19 (Simonyan and Zisserman, 2015).

#### 2.4.5 Classification performance

To evaluate the performance of each classifier, we computed the accuracy, defined as the percentage of correctly classified items over the total amount of items in the test set. Successful decoding must provide a performance at least above chance level, which is the probability of a correct prediction when randomly choosing one of the options. Thus, for two-classes problem a chance level is 50% and for four-classes problem a chance level is 25%. For the multiclass problem (four classes problem) we also provided a confusion matrix (Markoulidakis et al., 2021) to understand how classes are related to each other or which class is easier to classify.

#### 2.4.6 Effect of learning

To investigate the effect of motor learning, we divided the data into two groups: *learning* – while the subjects are acquiring the sequence – and *learned* – at the end of the learning phase when the subjects already learnt the sequence. To define the *learning phase*, we compared the last block (when subjects was trained to perform the experimental task) versus all previous blocks, and to define the *learned phase* we compared the first block (when subjects are not yet trained) versus all following blocks (paired *t*-test, *α* = *p* < 0.05). Next, we applied the decoding strategy to a subset of trials to evaluate the sensitivity of the decoding on the change in underlying neural pattern. Specifically, we generated two classifiers, one trained on *learning phase*, and one trained on the *learned phase*. Both classifiers were tested against learning and learned test trials. Performances are compared (paired *t*-test, α *= p* < 0.05).

Differences between the temporal latent sources of the learning and learned phases of the paradigm were quantified by non-parametric cluster statistics in terms of *F*-values. The similarity across spectral patterns was quantified in terms of scalar product among spatial patterns coefficients.

The code is available at: https://github.com/1101AlexZab1011/FingerMovementDecoder

## 3 Results

### 3.1 Behavioral performance

**Figure 3:**
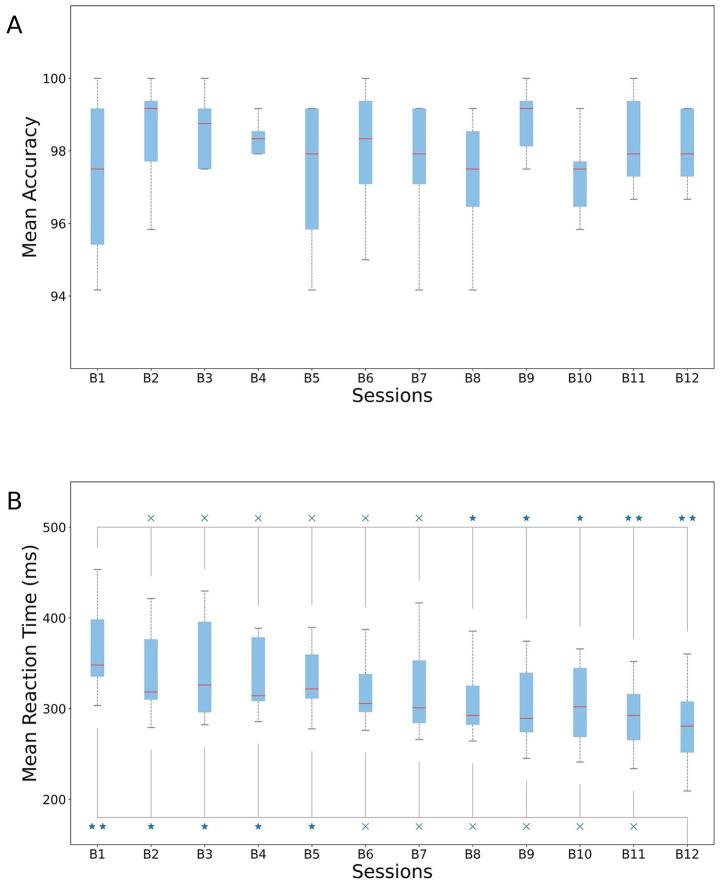
Behavioral performance in the SRTT. **(A)** Mean accuracy and **(B)** mean reaction times across subjects for each of the 12 blocks. Upper line: paired *t*-test results between 1st block and all the others. Bottom line: paired *t*-test results between 12th block and all the previous. *p < 0.05 and **p < 0.01.

We first quantified behavioral responses across the 12 blocks of our experimental design. No significant differences in accuracy (1st vs. 12th block, paired *t*-test, *p* = 0.80) were found, which was on average 97.48 ± 2.51% (Figure 3A). The profile of the response times mirrored the learning effect, with slowest responses in the first blocks and fastest responses in the last blocks of the experimental paradigm. We observed faster reaction times for blocks 8–12 compared to block 1 (Figure 3B) and in block 12 compared with block 1–5 (Figure 3B; Supplementary Table S1).

From the results, we concluded that subjects were still in the learning phase until the end of block 5, and that they might have already learned the sequence by the start of block 8. To maximize the separation between these two phases, we subsequently considered Blocks 1–3 as the *learning* phase and Blocks 10–12 as the *learned* phase.

### 3.2 Left vs. right-hand movement decoding

**Figure 4:**
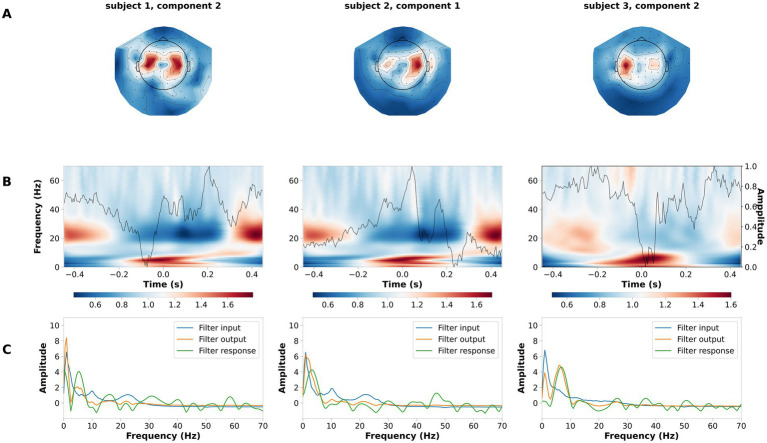
Example of spatial temporal and spectral patterns for left- vs. right-hand movement decoding. Each column is a representative component of the LF-CNN featuring: **(A)** spatial patterns; **(B)** latent sources: time resolved average (gray line) and average of the single trial time frequency representation obtained with Morlet complex wavelet (background). Time = 0 indicates response time; **(C)** FIR filter input (blue), filter response (green), and filter output (orange).

We validated the performance of LF-CNN on the laterality of the button press, between left- and right-hand side. Across participants, the average performance of LF-CNN was above 95% (98.5 ± 1.2%). Typical patterns for the spatial, spectral and temporal filters optimized by the LF-CNN are depicted in Figure 4. The first layer of LF-CNN returns spatial patterns with focal maxima in central areas (Figure 4A), therefore mirroring the relevance of motor activity. The second layer of LF-CNN returns time-frequency power patterns (Figure 4B), with modulations in alpha (8–15 Hz), beta (15–30 Hz) and gamma (>30 Hz) bands around the button press (t = 0) (Mohseni et al., 2020). Interestingly, the spectral properties of the temporal filter extracted from the second layer (Figure 4C, green trace) shows the prominent contribution of low frequency power. Therefore, the LF-CNN not only provides high classification performance, but also returns physiologically interpretable patterns in spatial, temporal and spectral domains.

### 3.3 Four finger movement decoding

Next, we validated the LF-CNN on the classification of four finger movements. We obtained a test classification accuracy of 82.0 ± 0.3%, which was in range with the performance of more complex architecture as FBCSP ShallowNet, EEGNet and Vgg19 (Table 1).

**Table 1:** Performance (test accuracy), runtime (training, validation and testing for all subjects, in seconds) and number of parameters for comparative deep-learning approaches for the decoding of finger movement in a motor sequence learning task.

Subjects	LFCNN	FBCSP	EEGNet	VGG19
1	0.79	0.79	0.80	0.78
2	0.85	0.82	0.83	0.84
3	0.81	0.79	0.84	0.83
4	0.88	0.87	0.90	0.91
5	0.85	0.79	0.83	0.81
6	0.81	0.79	0.84	0.79
7	0.86	0.84	0.85	0.90
8	0.81	0.76	0.83	0.86
Accuracy	0.83 ± 0.03	0.81 ± 0.03	0.84 ± 0.03	0.84 ± 0.05
Runtime	20 ± 1.35	126.69 ± 5.48	57.22 ± 6.04	1,307 ± 19.4
n_params	17,092	216,452	14,268	1,072,356

Additional performance metric and comparative statistics are provided in Supplementary Tables S4–S6.

Importantly, given the simpler architecture and the lower number of parameters to train, LF-CNN reached this level of accuracy in a runtime which was one order magnitude faster than FBSCP and around two orders of magnitude faster than Vgg19. Interestingly, the runtime was also superior to EEGNet, despite the comparable number of parameters to be trained. Runtimes reported here were evaluated on a Workstation, operative system Ubuntu 22.04.1 LTS, Processor Intel@ Xeon(R) CPU 2.10 GHz, RAM 128 GB.

We controlled for the distribution of misclassified trials reporting the performance in a confusion matrix (Table 2), which shows that the tendency to misclassify one finger with the ipsilateral finger is higher than with a contralateral finger. Beside the highly overlapping anatomical representation of ipsilateral fingers (Beisteiner et al., 2001; Liao et al., 2014), the correct performance holds stable at or above 80%, with chance level at 25% for the four fingers and at 50% for the ipsilateral hand.

**Table 2:** Confusion matrix for four finger movement decoding by LF-CNN.

	Predicted RM	Predicted RI	Predicted LM	Predicted LI
Actual RM	83 (7)	19 (4)	1 (1)	1 (1)
Actual RI	14 (8)	83 (5)	1 (1)	2 (1)
Actual LM	1 (1)	1 (1)	86 (7)	12 (4)
Actual LI	4 (1)	4 (1)	13 (6)	82 (4)

Predictions (%) as average (standard deviation) across all eight participants. RM, right middle, RI, right index, LM, left middle, LI, left index.

**Figure 5:**
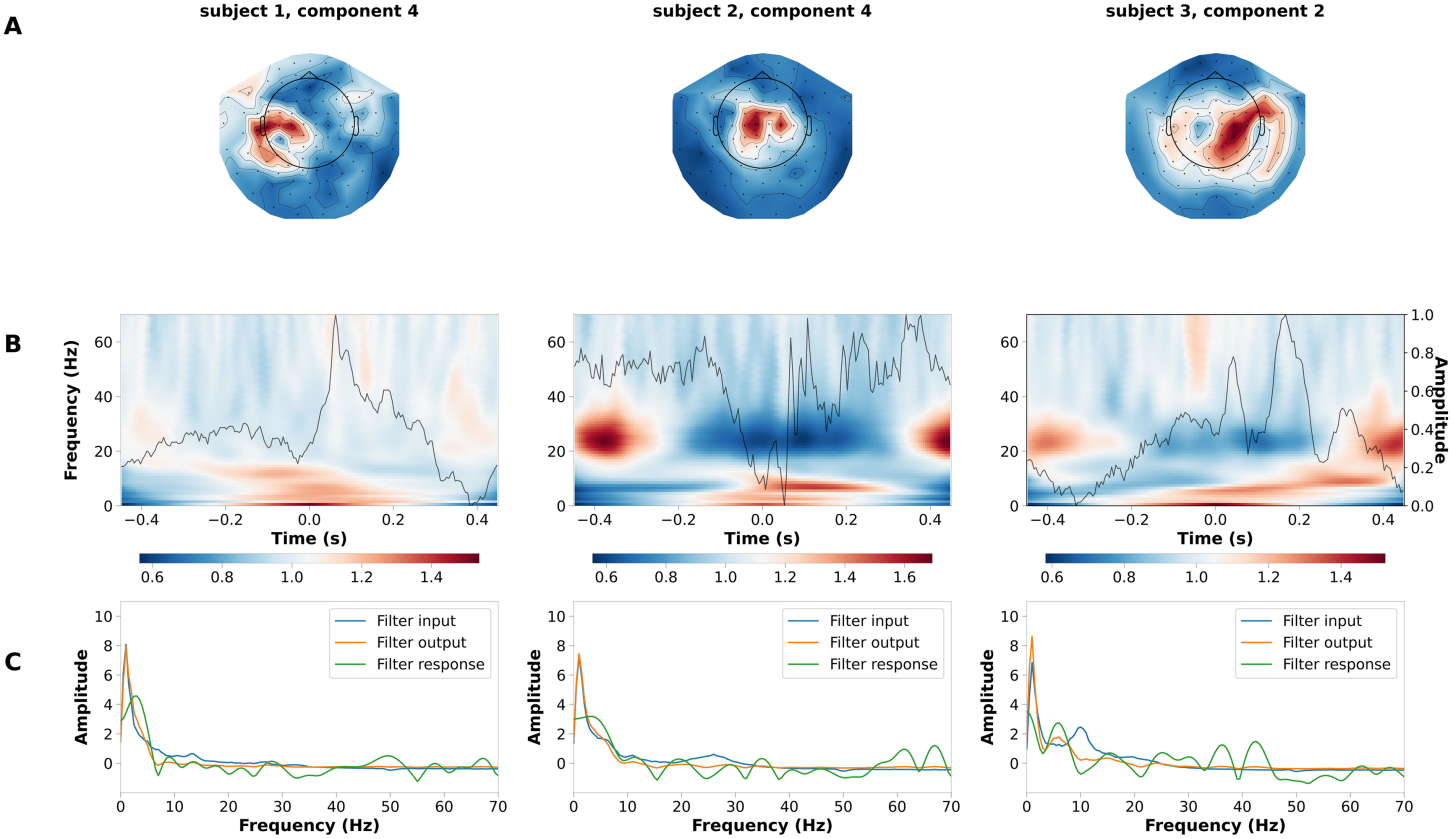
Example of spatial temporal and spectral patterns for four finger movement decoding. Each column is a representative component of the LF-CNN. **(A)** spatial patterns; **(B)** latent sources: time resolved average (gray line) and average of the single trial time frequency representation (background). Time = 0 indicates response time; **(C)** FIR filter input (blue), filter response (green), and filter output (orange).

**Figure 6:**
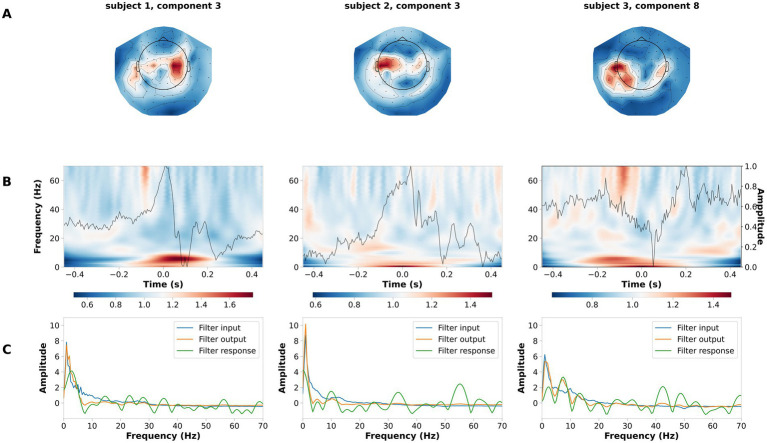
Example of spatial temporal and spectral patterns for ipsilateral fingers decoding. Each column is a representative component of the LF-CNN. **(A)** spatial patterns; **(B)** latent sources: time resolved average (gray line) and average of the single trial time frequency representation (background). Time = 0 indicates response time; **(C)** FIR filter input (blue), filter response (green), and filter output (orange).

The LF-CNN decoder provides physiologically interpretable spatial, temporal and spectral patterns. LF-CNN first layer returns spatial patterns with highest values over central areas (Figure 5A), while the second layer shows marked power modulation in the alpha and beta range (Figure 5B) and a temporal filter response with a low-pass spectral profile (Figure 5C). We performed an ablation study by systematically suppressing the contribution of spatial, temporal and fully connected layers. This revealed a prominent role of the spatial and fully connected layer (Supplementary Table S9). We tested the relevance of low frequencies for decoding high-pass filtering the data at 5 Hz, and observed a decrease in the LF-CNN accuracy to 0.64 (± 0.03). We tested the temporal sensitivity of the decoding and observed maximal performance for a 300 ms window centered at 50 ms after the button press (accuracy 78.67 ± 5.53%, Supplementary Figure S1). As an additional control, we also trained LF-CNN for classification of finger movements within the left hand and within the right hand. These models provided an accuracy 77.95 ± 5.06% for within left-hand movements 82.88 ± 5.19% for within right-hand movements (Figure 6).

### 3.4 MEG—cognitive status

The SRTT is designed to induce sequence learning, with the motor planning strategy changing between the early and later blocks of the experiment. To evaluate the effect of learning on the decoding performance, we generated two sets of models, one trained on the *learning* phase (blocks 1–3) and one on *learned* phase (blocks 10–12, see 3.1). We then tested these two sets of models on test data from both phases.

**Figure 7:**
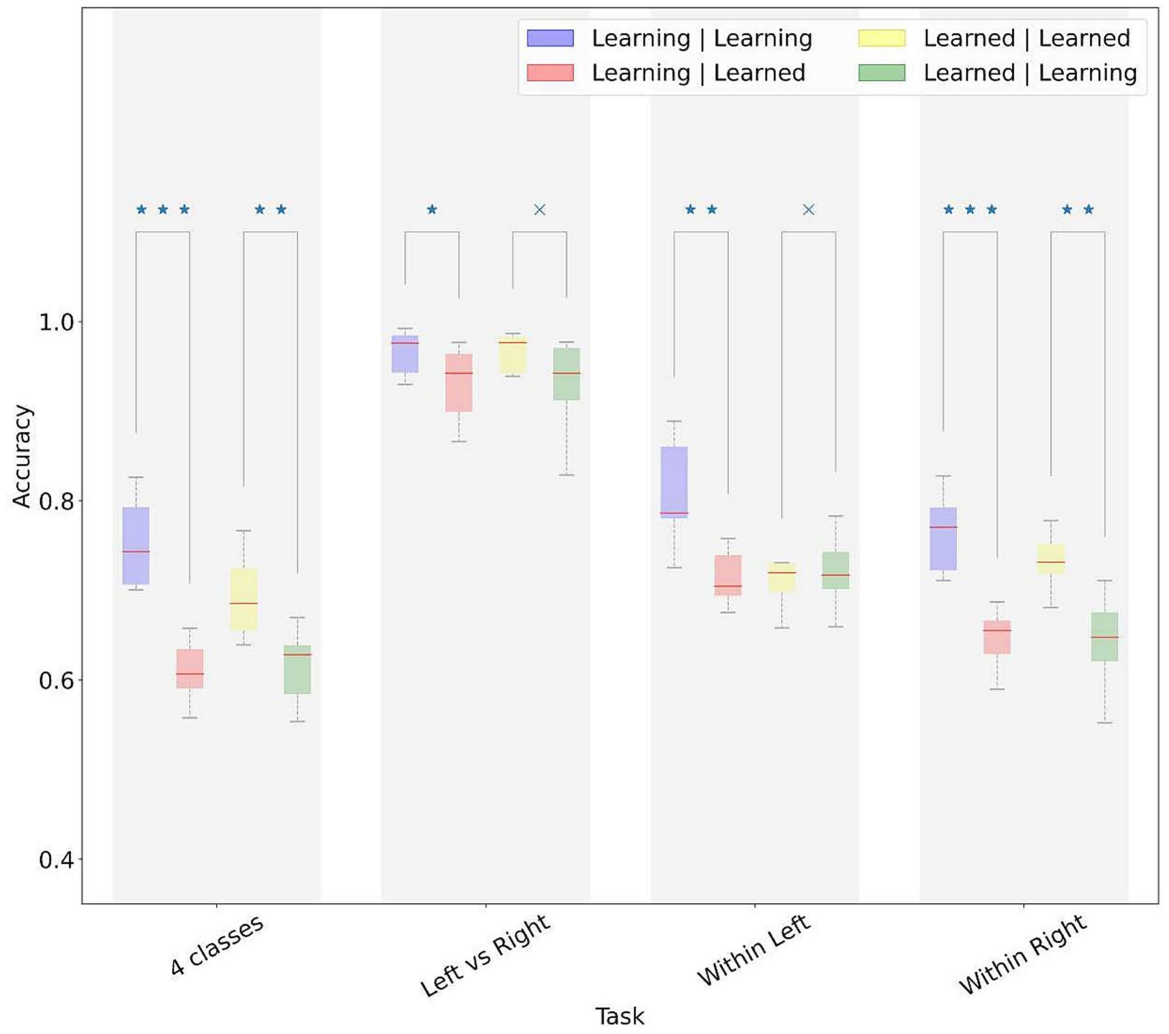
Performance of models across learning and learned phases of the SRTT paradigm. Model performances were averaged across participants. In the legend, the first term refers to the phase (learning vs. learned) of the training data, and the second term refers to the phase of the test data. x, not significant difference; *p-value < 0.05, **p-value < 0.01, ***p-value < 0.001.

In the finger movement decoding, both models performed better on test trials extracted from within the same phase (*learning* or *learned*) used for model training (Figure 7). The effect of phase on decoding was less visible in the hand decoding. For decoding within the same hand, we observed a significant phase effect for the right but not left-hand.

**Figure 8:**
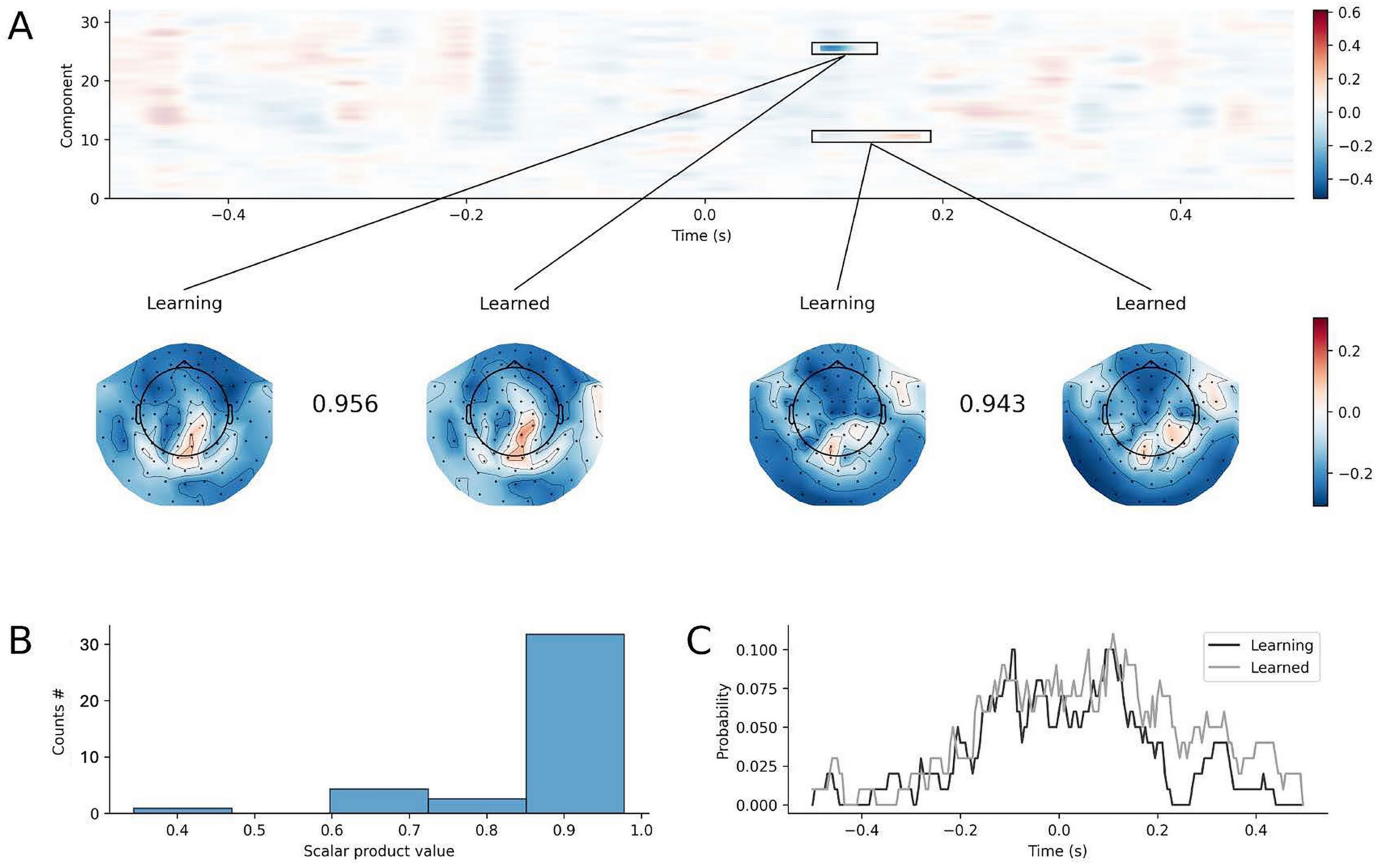
Difference in decoding models built from the learning and learned phases of the SRTT paradigm. **(A)** A representative example of the differences (*F*-values) between the temporal latent sources obtained by feeding a decoder trained on blocks 1–3 with data from blocks 1–3, and temporal sources obtained by feeding the same decoder trained on blocks 1–3 with data from blocks 10–12. Significant differences surviving non-parametric cluster permutation test are highlighted and spatial patterns topographies are shown (the scalar product between the spatial patterns measures their similarity). **(B)** Scalar product of spatial patterns of significant clusters obtained from the difference between the latent temporal sources of the learning (block 1–3) and learned (block 10–12) phases of the SRTT paradigm, obtained using both the decoders trained on learning (block 1–3) and learned (block 10–12) phases (x-axis: scalar product value: y-axis: count of scalar products for each bin). **(C)** Percentage of statistically significant differences in the temporal pattern of the 32 latent sources for models trained during the learning (black) and learned (gray) phase.

To explain the difference in decoding performance, we investigated the difference in latent sources obtained by feeding trials from the learning phase and the learned phase of the SRTT to the four-finger decoder trained on learning phase. Figure 8A shows the comparison between the obtained latent sources over time. Across subjects, only a minor number of temporal latent sources showed significant difference (range 1 to 4 out of 32 estimated components in each subject). In those components showing difference in the temporal trend, the spatial pattern was highly similar as shown by the scalar product in the inset of Figure 8A, and in the distribution of the scalar product computed for all significant clusters in Figure 8B. The difference in the temporal latent sources obtained injecting data from the learning vs. learned phases was distributed mostly in the – 200 to + 200 ms interval around button press (Figure 8C: two curves for two decoders trained on the learning and learned phases). Therefore, the difference in the properties of the decoders were minimal, distributed on different components, and variable across individuals.

## 4 Discussion

In this study, we showed that it was possible to reliably decode finger movements from non-invasive neurophysiological data in healthy participants performing the SRTT, operationalized as a motor learning task. Through our compact deep learning model, the LF-CNN, we showcased high classification accuracy and physiologically interpretable results.

The LF-CNN accuracy performance was comparable to that of more complex deep learning architectures, with significantly reduced training time. This was due to a lower number of parameters to be optimized (Schirrmeister et al., 2017; Simonyan and Zisserman, 2015) and without the need of a dense layer, thus reducing time-consuming convolutions (Lawhern et al., 2018). Specifically, the LF-CNN was able to decode dynamically changing spatio-temporal parameters in approximately 20 s compared to almost 60 s of EEGNet (Table 1). This decoding efficiency and performance gain can improve the timing of essential information in applied rehabilitation situations, whereby feedback on cortical contributions to motor learning is essential. In the next sections, we go into the details of the decoding structure from our model and their role in understanding of motor and cognitive processing.

### 4.1 Finger movement decoding and motor learning

In the current study, the LF-CNN approach appears to utilize both spatial and temporal filters, represented by the weights of the trained network layers, to support the decoding of finger movements. While these weights may not directly reflect neural activity (Haufe et al., 2014; Petrosyan et al., 2021), they can offer insights into the underlying signal structure. The resulting patterns may inform a generative model that characterizes the potential relationship between sensor-level recordings and neural sources (Cui et al., 2022; Petrosyan et al., 2021).

The first convolutional layer applies spatial filters across the MEG sensor array, performing a one-dimensional convolution to project the high-dimensional MEG signals into a lower-dimensional latent space. This projection captures dominant spatial features related to neural sources, significantly reducing the complexity of the data while preserving key information. Notably, the spatial patterns revealed prominent activity in sensorimotor cortices, areas known to be critical for motor planning and execution (Cheyne, 2013; Pfurtscheller and Lopes da Silva, 1999). These regions exhibited event-related synchronization (ERS) and desynchronization (ERD) in the alpha and beta frequency bands that are well-established markers of motor preparation and execution (Kilavik et al., 2013; Pfurtscheller and Neuper, 2003).

The second convolutional layer uses temporal filters that behave like univariate autoregressive (AR) models, allowing for direct interpretation in the frequency domain. These temporal filters decompose the signal into oscillatory components, forming spectral patterns that reflect the dynamics of brain rhythms associated with motor control. The learned filters predominantly exhibited low-pass characteristics, emphasizing slow oscillations (<30 Hz) that have been repeatedly implicated in motor behavior and skill acquisition (Engel and Fries, 2010; Tan et al., 2016).

Our key contribution of this study is the ability to distinguish movements of the ipsilateral index and middle fingers with a mean classification accuracy of 82%, that is performing as well as current popular CNNs with a significant reduction of computation time. Early work demonstrated somatotopic segregation of individual fingers in the somatosensory cortex (Kleinschmidt et al., 1997), a finding later refined through modelling approaches using Gaussian receptive fields (Schellekens et al., 2018). Invasive recordings using ECoG revealed distinct broadband spectral patterns for individual finger movements and movement trajectories (Miller et al., 2009), leading to highly accurate finger decoding for prosthetic control (up to 92%; see Hotson et al. (2016)). However, translating these results to non-invasive modalities like EEG and MEG typically reduces classification performance (Liao et al., 2014). Still, notable progress has been made: Quandt et al. (2012) initiated non-invasive finger decoding efforts, while more recent approaches, such as wavelet-based feature extraction with SVM-RBF classifiers (Alazrai et al., 2019) and optimized channel selection strategies (Gannouni et al., 2020) have achieved accuracies around 81–86% for finger classification. Our findings build on this momentum, demonstrating robust decoding of fine motor learning that dynamically progresses in a non-invasive MEG setting.

Our decoding results also showed higher accuracy when using broadband MEG data compared to focusing solely on the alpha/beta range, which are considered the prominent motor-related frequency bands. Notably, restricting analysis to alpha and beta bands did not improve performance. This aligns with prior findings showing that broadband features outperform narrowband (alpha/beta) approaches in non-invasive settings (Lee et al., 2022; Liao et al., 2014; Quandt et al., 2012). While some studies using alpha/beta power achieved moderate accuracy (Alazrai et al., 2019; Gannouni et al., 2020), broadband analysis consistently yielded better results, including in the present work. These findings suggest that low-frequency broadband activity captures richer, spatially localized information relevant to finger motor planning and execution.

Previous approaches (Alazrai et al., 2019; Gannouni et al., 2020; Lee et al., 2022; Quandt et al., 2012) typically employ a two-step pipeline: feature preselection followed by classification using standard machine learning algorithms. However, these methods often lack physiological interpretability of the selected features, potentially limiting their insight into neural processes. In contrast, our model learns spatial and temporal (spectral) features directly from the data, producing interpretable patterns that reflect underlying neural activity. More broadly, this approach could extend to decoding more complex motor tasks, such as wrist extension/flexion (Vuckovic and Sepulveda, 2008), differentiation of thumb, fist, and individual finger movements (Javed et al., 2017), or distinguishing grasp types like palmar, lateral, and pinch (Jochumsen et al., 2016). Enhancing finger decoding could also benefit BCI applications focused on movement trajectories like 2D finger pointing (Toda et al., 2011) to 3D hand movements (Korik et al., 2018). This could lay the groundwork for precise kinematic decoding of speed, velocity, and complex tasks like center-out reaching or drawing (Bradberry et al., 2009; Kobler et al., 2019; Lv et al., 2010) could be extended to neuroprosthetics.

### 4.2 Sensitivity to the cognitive state changes and implications for future work

Although deep learning methods typically require large datasets, our study demonstrates that LF-CNN can achieve high classification accuracy even with a limited number of trials. Notably, the model was sensitive to learning-related changes within the SRTT paradigm. By comparing decoder patterns between the early *learning* and later *learned* phases, we investigated intra-subject variability, with minimal differences in latent sources and spatial distribution. On the other hand, the high performance for each individual and the low level of generalization across subjects highlights the model’s ability to capture subject-specific neural dynamics associated with motor learning. This underscores the model’s capacity to detect subtle, individualized changes in brain activity, which is an essential feature for probing the neurophysiological mechanisms of learning and behavior. Moreover, its sensitivity suggests strong potential for broader applications in identifying context-specific variations in neural activity. This study represents a stride towards the development of adaptive algorithmic approaches for tackling intricate decoding challenges and elucidating the neurophysiological mechanisms of associated cognitive processes in complex motor tasks. The combination of spatial and spectral patterns supports a data-driven decoding model that captures how different brain rhythms and regions contribute to motor performance.

In the context of motor learning, this decoding approach may offer several valuable perspectives. For instance, as individuals acquire or refine motor skills, changes in the spatial distribution and strength of ERD/ERS patterns have been observed, potentially reflecting cortical reorganization (Brunner et al., 2024; Dayan and Cohen, 2011). The LF-CNN may be sensitive to such shifts in spatial representations, as suggested by the low level of generalization across subjects (Supplementary Table S8). While this accounts for subject specific distribution of the motor signaling detectable through MEG, it also provides insight into learning-related changes in the cortex (Borràs et al., 2025). Secondly, by identifying task-relevant and isolating specific frequency bands frequencies that improve movement decoding, the model reveals the rhythmic components most involved in motor learning (e.g., increased beta-band desynchronization during skilled performance). Thirdly, the decoding accuracy of motor-related states can serve as a proxy for monitoring learning progress and changes in cognitive states. As a subject becomes more proficient, the model’s ability to distinguish motor intentions or executions may improve, reflecting more robust and consistent neural representations. Lastly, the extracted spatial and spectral patterns can inform neurofeedback or brain-computer interface (BCI) applications aimed at enhancing motor learning by setting neurofeedback targets alongside timely feedback (Soekadar et al., 2015; Vidaurre et al., 2011).

Ultimately, the model’s sensitivity to neurophysiological variations holds promising implications for neurorehabilitation, which could be tested in stroke recovery. Fine motor impairments are common after stroke, with tools like the 9-Hole Peg Test routinely used to assess motor coordination (Oxford Grice et al., 2003). The ability to decode specific finger movements enables objective, real-time monitoring of recovery progress (Guger et al., 2017; Khan et al., 2020). Previous studies have shown the effectiveness of BCI-driven interventions in improving motor control in stroke patients (Ramos-Murguialday et al., 2013; Romero-Laiseca et al., 2020). For example, detecting neural activation associated with index and middle finger movements may indicate cortical-level recovery, even in the absence of overt motion. Repeated engagement of these neural patterns over time could signal positive trends in fine motor function and the prediction of motor kinematics with CNNs (Borra et al., 2023). Furthermore, accurate decoding of finger movements can support the precise control of prosthetic devices like enabling pinch or grasp functions (Jain and Kumar, 2025) and thereby providing a valuable tool for restoring functional independence in stroke patients. These findings align with our approach, which provides a promising avenue for enhancing neurorehabilitation through precise neural decoding and motor control (Tian et al., 2025).

## 5 Summary

We used a physiologically interpretable convolutional neural network (LF-CNN) to decode motor finger movements during the learning of the Sequential Reaction Time Task (SRTT), where participants pressed specific buttons in response to visual cues showing a repeating sequence. The LF-CNN effectively identified distinct activation patterns across spatial, temporal, and spectral domains. Compared to more complex models, it offered a notable advantage in computational efficiency, with significantly reduced runtime, while maintaining interpretability of learned weights. Importantly, the LF-CNN was sensitive to different stages of motor learning, enabling individualized monitoring of learning dynamics for diagnostic purposes. In conclusion, the LF-CNN enables a principled decomposition of MEG signals into spatial and temporal components that are both interpretable and behaviorally relevant. This approach not only enhances decoding performance but also offers a powerful framework for investigating the neural basis of motor learning.

## Data Availability

The raw data supporting the conclusions of this article will be made available by the authors, without undue reservation.
