# Supplementary material for: Convolutional neural networks decode finger movements in motor sequence learning from MEG data
Source: Front Neurosci. 2025 Sep 9;19:1623380. doi: 10.3389/fnins.2025.1623380 (PMC12454436; doi:10.3389/fnins.2025.1623380)
Supplement: Supplementary file 1 [file Data_Sheet_1.PDF]

Supplementary material

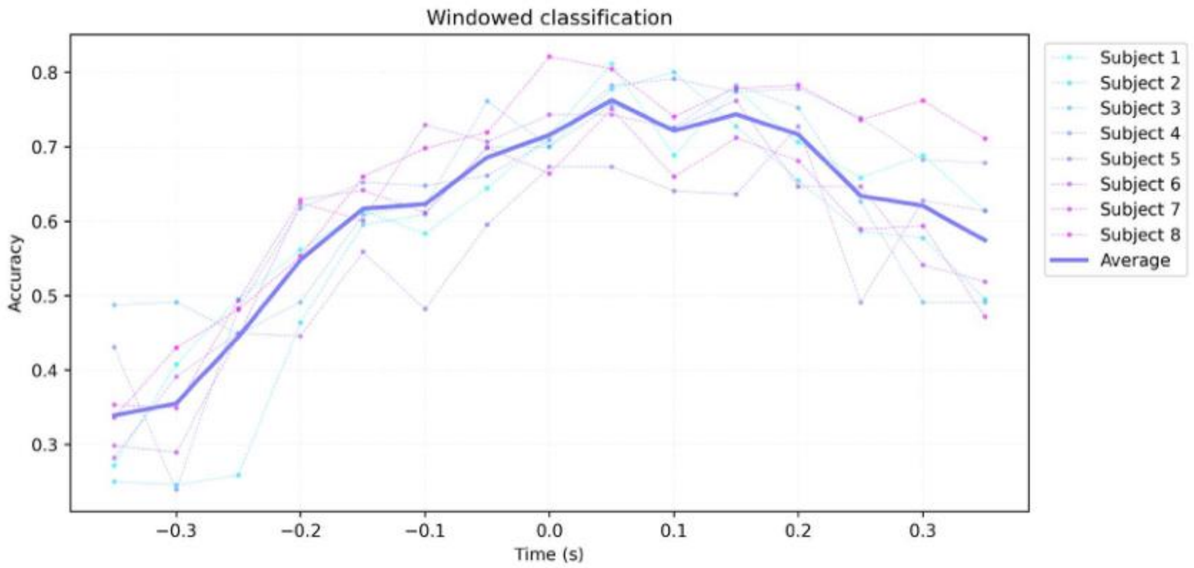

Figure 1S. Temporal sensitivity analysis on the four fingers decoding. We iteratively ran the decoding procedure considering the data included in a 300 ms window moving in steps of 50 ms. We observed the maximal performance averaged across subjects at 50 ms after the button press (accuracy 78.67 ± 5.53 %).

**Accuracy**	**Experimental Block**											
**No.**	**1**	**2**	**3**	**4**	**5**	**6**	**7**	**8**	**9**	**10**	**11**	**12**
1	100.00	100.00	99.17	99.17	99.17	100.00	95.83	98.33	97.50	96.67	96.67	99.17
2	99.17	98.33	100.00	100.00	99.17	100.00	99.17	99.17	100.00	99.17	100.00	99.17
3	99.17	95.83	98.33	98.33	95.83	98.33	98.33	99.17	99.17	97.50	100.00	96.67
4	99.17	99.17	99.17	98.33	98.33	98.33	97.50	97.50	99.17	98.33	99.17	97.50
5	89.17	91.67	85.83	90.83	94.17	95.83	94.17	94.17	92.50	91.67	93.33	93.33
6	95.83	99.17	99.17	98.33	97.50	99.17	99.17	97.50	98.33	95.83	97.50	99.17
7	94.17	100.00	97.50	96.67	95.83	95.00	97.50	95.83	99.17	97.50	98.33	97.50
8	95.83	99.17	97.50	98.33	99.17	97.50	99.17	96.67	100.00	97.50	97.50	98.33

**Reaction Times (ms)**	**Experimental Block**											
**No.**	**1**	**2**	**3**	**4**	**5**	**6**	**7**	**8**	**9**	**10**	**11**	**12**
1	330	313	317	311	317	306	300	274	245	241	234	209
2	453	362	389	376	351	387	351	386	374	365	334	325
3	383	420	430	387	389	373	360	365	347	366	352	360
4	348	321	335	302	326	305	285	285	275	300	309	288
5	303	279	282	314	294	282	266	264	281	263	265	266
6	444	421	416	389	387	326	417	312	337	338	265	157
7	337	300	282	286	278	276	281	285	270	271	276	273
8	348	316	300	315	317	301	301	300	297	304	310	302

Table 1S. Accuracy and Reaction times for each subject (y-axis) and experimental block (x-axis).

Subject	RM	RI	LM	LI	total
1	349	316	336	319	1320
2	337	317	341	327	1322
3	344	344	340	341	1369
4	343	343	345	349	1380
5	332	346	355	347	1380
6	332	320	338	320	1310
7	354	351	352	353	1410
8	350	348	340	336	1374

Table 2S. Number of trials utilized per subject in the four finger decoding. RM = right middle, RI = right index, LM = left middle, LI = left index.

**Parameter**	**value**
Number of latent sources	32
Temporal filter length	50
Learning rate	3.00E-04
l2-penalty	0.000001
Pooling	max
Pooling factor	10
Drop-out coefficient	0.4
Input layer link function	identity
Hidden layer link function	elu
Output nonlinearity	softmax
Number of dense hidden layers	1
Optimizer	Adam
Early Stopping:	
minimum delta in the loss	1e-6
patience (minimum number of epochs with no improvement)	3

Table 3S. LF-CNN Hyperparameter settings.

Subject	Model	Accuracy	Precision	Recall	F1	kappa
1	VGG19	0.777	0.777	0.782	0.777	0.702
1	EEGNet	0.800	0.804	0.800	0.799	0.733
1	FBCSP	0.786	0.782	0.787	0.781	0.713
1	LFCNN	0.786	0.786	0.782	0.782	0.713
2	VGG19	0.841	0.843	0.843	0.841	0.788
2	EEGNet	0.832	0.834	0.832	0.832	0.776
2	FBCSP	0.823	0.818	0.813	0.815	0.761
2	LFCNN	0.850	0.851	0.852	0.850	0.800
3	VGG19	0.829	0.829	0.829	0.829	0.772
3	EEGNet	0.838	0.837	0.836	0.836	0.783
3	FBCSP	0.785	0.796	0.791	0.784	0.714
3	LFCNN	0.811	0.808	0.818	0.810	0.748
4	VGG19	0.909	0.909	0.908	0.908	0.878
4	EEGNet	0.896	0.899	0.896	0.895	0.861
4	FBCSP	0.865	0.868	0.864	0.865	0.820
4	LFCNN	0.878	0.881	0.881	0.878	0.838
5	VGG19	0.813	0.814	0.813	0.813	0.750
5	EEGNet	0.826	0.824	0.830	0.824	0.768
5	FBCSP	0.791	0.790	0.796	0.792	0.719
5	LFCNN	0.852	0.854	0.852	0.850	0.802
6	VGG19	0.794	0.794	0.795	0.794	0.725
6	EEGNet	0.844	0.851	0.847	0.847	0.791
6	FBCSP	0.794	0.791	0.792	0.791	0.724
6	LFCNN	0.812	0.809	0.813	0.810	0.748
7	VGG19	0.898	0.897	0.897	0.897	0.864
7	EEGNet	0.851	0.855	0.852	0.851	0.802
7	FBCSP	0.838	0.840	0.841	0.840	0.783
7	LFCNN	0.855	0.857	0.858	0.855	0.807
8	VGG19	0.860	0.859	0.858	0.858	0.813
8	EEGNet	0.834	0.835	0.833	0.833	0.779
8	FBCSP	0.764	0.765	0.766	0.764	0.686
8	LFCNN	0.812	0.808	0.816	0.810	0.748

Table 4S. Accuracy, Precision, Recall, F1, Cohen's kappa for each subject and model (see Methods section for details) for the four fingers decoding.

metric	F Value	Num DF	Den DF	Pr > F
**accuracy**	5.7547	3	21	0.0049
**precision**	6.0586	3	21	0.0039
**recall**	6.7419	3	21	0.0023
**F1**	6.7335	3	21	0.0023
**kappa**	6.0522	3	21	0.0039

Table 5S. Repeated measures ANOVA on testing differences in performance metrics.

	Model 1	Model 2	t-statistic	p-value (uncorrected)	p-value (Bonferroni)
**accuracy**	VGG19	EEGNet	0.002	0.999	1.000
	VGG19	FBCSP	2.845	0.025	0.149
	VGG19	LFCNN	0.709	0.501	1.000
	**EEGNet**	FBCSP	4.388	0.003	0.019
	EEGNet	LFCNN	1.034	0.336	1.000
	FBCSP	**LFCNN**	-3.807	0.007	0.040
**precision**	VGG19	EEGNet	-0.209	0.840	1.000
	VGG19	FBCSP	3.032	0.019	0.114
	VGG19	LFCNN	0.753	0.476	1.000
	**EEGNet**	FBCSP	5.046	0.001	0.009
	EEGNet	LFCNN	1.209	0.266	1.000
	FBCSP	LFCNN	-3.614	0.009	0.051
**recall**	VGG19	EEGNet	-0.018	0.986	1.000
	VGG19	FBCSP	3.163	0.016	0.095
	VGG19	LFCNN	0.675	0.521	1.000
	**EEGNet**	FBCSP	4.879	0.002	0.011
	EEGNet	LFCNN	0.975	0.362	1.000
	FBCSP	**LFCNN**	-3.965	0.005	0.033
**F1**	VGG19	EEGNet	0.005	0.996	1.000
	VGG19	FBCSP	3.229	0.014	0.087
	VGG19	LFCNN	0.870	0.413	1.000
	**EEGNet**	FBCSP	4.838	0.002	0.011
	EEGNet	LFCNN	1.155	0.286	1.000
	FBCSP	**LFCNN**	-4.068	0.005	0.029
**kappa**	VGG19	EEGNet	-0.011	0.992	1.000
	VGG19	FBCSP	2.937	0.022	0.131
	VGG19	LFCNN	0.752	0.477	1.000
	**EEGNet**	FBCSP	4.607	0.002	0.015
	EEGNet	LFCNN	1.094	0.310	1.000
	FBCSP	**LFCNN**	-3.793	0.007	0.041

Table 6S. Post-hoc analysis providing parirwise comparision of differences in model performance for different metrics. When significant differences are detected (Bonferroni corrected *p*-value highlighted in green). Better performing models are highlighted in bold.

Subject	Test_acc
1	0.75
2	0.60
3	0.69
4	0.86
5	0.72
6	0.81
7	0.82
8	0.73
**Accuracy**	0.74 ± 0.07
**Runtime**	15.7 ± 2.67

Table 7S. Classification acccuracy and runtime (in seconds) for the four fingers decoding on simulus-locked trials.

Train \ Test	1	2	3	4	5	6	7	8
1	0.79	0.40	0.36	0.41	0.30	0.43	0.39	0.38
2	0.36	0.85	0.49	0.51	0.48	0.48	0.57	0.38
3	0.37	0.49	0.81	0.44	0.42	0.42	0.57	0.39
4	0.35	0.45	0.44	0.88	0.36	0.48	0.44	0.33
5	0.37	0.54	0.40	0.33	0.85	0.38	0.59	0.38
6	0.41	0.49	0.45	0.34	0.35	0.81	0.43	0.36
7	0.39	0.52	0.41	0.48	0.45	0.41	0.86	0.39
8	0.39	0.45	0.43	0.45	0.34	0.48	0.49	0.81

Table 8S. Accruacy of cross-subject generalisation for the four-fingers decoder. Train data and test data from different subject (1-8).

spatial demixing	temporal convolution	fully connected	test accuracy
			0.83 ± 0.03
■			78.26 ± 0.02
	■		80.66 ± 0.04
		■	81.69 ± 0.03
■	■		72.27 ± 0.04
■		■	57.29 ± 0.07
	■	■	69.29 ± 0.06

Table 9S. Ablation study on the LFCNN for the four-fingers decoder. In black are the frozen layers. The performance is expressed as the mean and standard deviation of accuracy across subjects.

	Trials	
	**[-300, 300]ms**	**[-500, 500]ms**
	0.72	0.79
	0.84	0.85
	0.80	0.81
	0.81	0.88
	0.80	0.85
	0.79	0.81
	0.83	0.86
	0.81	0.81
**Accuracy (mean)**	0.80	0.83
**Accuracy (std)**	0.03	0.03

Table 10S. Comparison of performance for the four fingers decoding with trials of [-300, 300] ms and [-500, 500] ms around the response time.
